# Doubly labelled water assessment of energy expenditure: principle, practice, and promise

**DOI:** 10.1007/s00421-017-3641-x

**Published:** 2017-05-15

**Authors:** Klaas R. Westerterp

**Affiliations:** Department of Human Biology, Maastricht University Medical Centre, PO Box 616, 6200 MD Maastricht, The Netherlands

**Keywords:** Doubly labelled water, Carbon dioxide production, Energy expenditure, Physical activity, Body composition, Energy requirement, Energy intake, Motion sensors, Body mass regulation

## Abstract

The doubly labelled water method for the assessment of energy expenditure was first published in 1955, application in humans started in 1982, and it has become the gold standard for human energy requirement under daily living conditions. The method involves enriching the body water of a subject with heavy hydrogen (^2^H) and heavy oxygen (^18^O), and then determining the difference in washout kinetics between both isotopes, being a function of carbon dioxide production. In practice, subjects get a measured amount of doubly labelled water (^2^H_2_^18^O) to increase background enrichment of body water for ^18^O of 2000 ppm with at least 180 ppm and background enrichment of body water for ^2^H of 150 ppm with 120 ppm. Subsequently, the difference between the apparent turnover rates of the hydrogen and oxygen of body water is assessed from blood-, saliva-, or urine samples, collected at the start and end of the observation interval of 1–3 weeks. Samples are analyzed for ^18^O and ^2^H with isotope ratio mass spectrometry. The doubly labelled water method is the indicated method to measure energy expenditure in any environment, especially with regard to activity energy expenditure, without interference with the behavior of the subjects. Applications include the assessment of energy requirement from total energy expenditure, validation of dietary assessment methods and validation of physical activity assessment methods with doubly labelled water measured energy expenditure as reference, and studies on body mass regulation with energy expenditure as a determinant of energy balance.

## Background

Measurement of whole-body metabolic rate is performed with direct calorimetry, based on the measurement of heat loss, or with indirect calorimetry, based on the measurement of oxygen consumption, carbon dioxide production, and urine-nitrogen loss for energy production from carbohydrate, protein, and fat. Measurement of energy expenditure with doubly labelled water is an innovative variant on indirect calorimetry, where energy expenditure is derived from the measurement of carbon dioxide production.

The doubly labelled water method for the measurement of energy expenditure was invented in the late 1940’s and early 1950’s (Table 1). Lifson et al. (1949) discovered that the oxygen in respiratory carbon dioxide is in isotopic equilibrium with the oxygen in body water. Two mice and a rat were forced to breathe oxygen enriched with heavy oxygen (^18^O). The heavy oxygen subsequently appeared in the animals’ body water and in expired carbon dioxide, leading to the conclusion that expired carbon dioxide derives its oxygen at least partly from body water. In a next experiment, animals were injected with ^18^O-enriched water, showing that the enrichment of ^18^O in body water and expired carbon dioxide were very similar and thus establishing isotopic exchange between body water and expired carbon dioxide. They performed subsequent studies on laboratory rats and mice to evaluate the potential of the invention to measure energy expenditure (Lifson and McClintock 1955). In 1966, they published a review paper, which forms the basis of the technique for studies of energy expenditure in free-ranging animals (Lifson and McClintock 1966).

**Table 1:** Key articles in development of the doubly labelled water method for the measurement of energy expenditure

1949	Discovery that the oxygen in respiratory carbon dioxide is in isotopic equilibrium with the oxygen in body water (Lifson et al. 1949)
1955	Description of the measurement of carbon dioxide production from isotopic analysis of body water (Lifson and McClintock 1955)
1964	Doubly labelled water assessment of the energy cost of flying in pigeons (LeFebvre 1964)
1966	Review paper on a model for doubly labelled water assessment of energy expenditure in free-ranging animals (Lifson and McClintock 1966)
1982	Validation of the doubly labelled water method for the measurement of energy expenditure in humans (Schoeller and Van Santen 1982)
1988	Workshop on standardization of the doubly labelled water method for the measurement of energy expenditure in humans (Prentice 1990)
1994	Symposium on advances in the doubly labelled water technique (Speakman and Roberts 1995)

Labelled water, water labelled with ^2^H and/or ^18^O, was initially applied to measure body composition. In a two-compartment model for body composition with the component fat-free mass and fat mass, water is an important constituent of fat-free mass (Pace and Rathbun 1945). Fat is free of water and the hydration of fat-free mass among healthy subjects is relatively constant. The hydration of fat-free mass decreases from 80% at birth to 73% at adult age Fomon et al. 1982). Thus, fat-free mass can be calculated from a total body water measurement with ^2^H and/or ^18^O dilution after drinking labelled water (Schoeller et al. 1980). Assessment of body composition by measuring total body water with isotope dilution is one of the two available methods, with the measurement of body volume with densitometry, for in vivo body composition measurement (Westerterp 2011). The assumptions on the hydration and the density of body components are derived from carcass analysis. Alternative body composition techniques, including skinfold thickness, body impedance, total body electrical conductivity, dual energy X-ray absorptiometry, and magnetic resonance imaging, are double indirect. The techniques are validated against the two indirect methods including isotope dilution. A recent example is the validation of the measurement of changes in body composition with bio impedance spectroscopy (Ellegård et al. 2016).

The first applications of the doubly labelled water method for the measurement of energy expenditure were restricted to small animals because of the high cost of ^18^O-enriched water. An early example is the measurement of the energy cost of flying in pigeons (LeFebvre 1964). My first application of the method was measurement of the energy budget of breeding House Martins, a 20-g bird spending most of the day on the wing (Bryant and Westerterp 1980). Advances in analytical devices and reductions in the costs of ^18^O-enriched water led to the suggestion that the method was useful for application to the field of human energy metabolism (Lifson et al. 1975). Subsequently, the first validation for the measurement of energy expenditure in humans was in 1982 (Schoeller and Van Santen 1982).

Schoeller and Van Santen (1982) administered approximately 10 g of ^18^O en 5 g ^2^H to four young adults, one female and three males, after collecting a baseline urine sample for measurement of the background enrichment of body water for the two isotopes. Subsequent urine samples were collected at 6 h after the dose, directly after equilibration of the isotope dose with total body water, and 14 days later at the end of the observation interval. During this interval, energy expenditure was calculated from energy balance by taking the sum of dietary intake and the change in body stores as reference. The energy expenditure from the doubly labelled water method differed from dietary intake plus change in body composition by 2 ± 6%. Thus, it was the first study showing the validity of the doubly labelled water method for the measurement of energy expenditure in unrestricted humans. Later, validation studies, comparing doubly labelled water-assessed energy expenditure with simultaneously measured energy expenditure in a respiration chamber, showed that the method is accurate and has a precision of 2–8% (Schoeller 1988).

In 1988, the method was already applied to humans in eight research centres and all users came together to discuss standardization of the method (Prentice 1990). Aspects included were isotope mass spectrometric analysis, calculation of isotopic pool sizes and flux rates, isotope fractionation corrections, changes in isotopic background, and converting carbon dioxide production to energy expenditure. A similar group presented further advances in the doubly labelled water technique in a workshop at the 78th annual Experimental Biology meeting (Speakman and Roberts 1995). Here, the protocol and analytical requirements for application of the doubly labelled water method were further defined and results were presented on a between laboratory comparison.

Since the mid 1980s, the use of the method has expanded enormously (Speakman 1998). The number of human studies as published in peer-reviewed journals rapidly increased to a steady level around 40 per year from 1995 onwards. It has become the gold standard for human energy requirement under daily living conditions (FAO/WHO/UNU 1985, 2004). Further applications include energy cost of clinical conditions (Van der Kuip et al. 2007), activity costs in relation to body mass (Prentice et al. 1996) and age (Speakman and Westerterp 2010), and under extreme conditions like endurance exercise (Westerterp et al. 1986; Cooper et al. 2011) and at high altitude (Westerterp et al. 1992).

## Principle

**Fig. 1:**
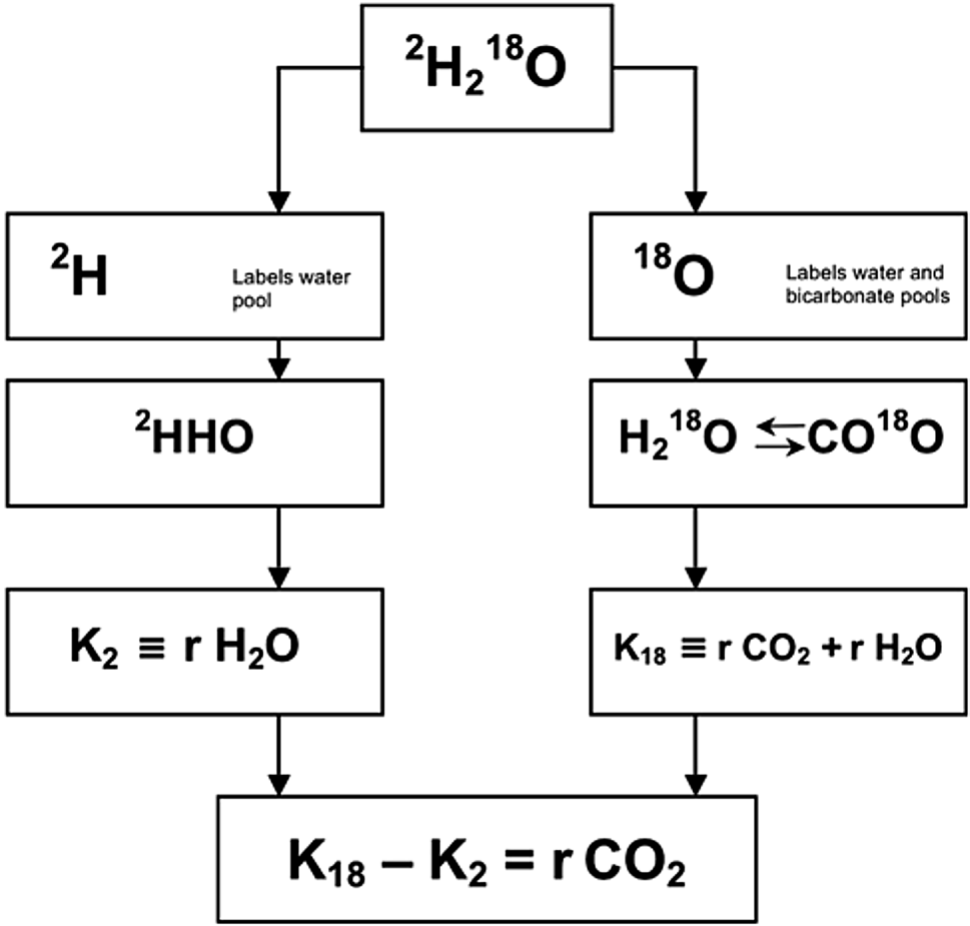
Principle of measurement of carbon dioxide production with doubly labelled water (^2^H_2_^18^O). After administration of water labelled with heavy oxygen (^18^O) and heavy hydrogen (^2^H), the two isotopes mix with the body water, where ^18^O exchanges with CO_2_ in the bicarbonate pools as well. Thus, the elimination rate of ^2^H (*K*_2_) is a measure for water loss (rH_2_O) and the elimination rate of ^18^O (*K*_18_) is a measure for rH_2_O plus carbon dioxide production (rCO_2_), and rCO_2_ = *K*_18_ − *K*_2_

The method to measure energy expenditure with doubly labelled water is based on the difference between the apparent turnover rates of the hydrogen and oxygen of body water as a function of carbon dioxide production. It involves enriching the body water of a subject with heavy oxygen (^18^O) and heavy hydrogen (^2^H) and then determining the difference in washout kinetics between both isotopes. The oxygen isotope is lost as water and as carbon dioxide, due to exchange in the bicarbonate pools. The hydrogen isotope is lost as water only (Fig. 1). In its simplest form, carbon dioxide production can be calculated with the formula:

$${\text{rCO}}_{2} ({\text{mol}}) = (N/ 2) \, (K_{ 1 8} - K_{ 2} ),$$

where rCO_2_ is the carbon dioxide production, *N* is the body water volume in mol, the value 2 is a constant reflecting that 1 mol CO_2_ removes two atoms of oxygen, and *K*_18_ and *K*_2_ are the elimination rates of heavy oxygen and heavy hydrogen, respectively.

The equation as presented above requires adjustment for incorporation of the two isotopes in other molecules than body water and carbon dioxide and for isotope fractionation when the isotopes are eliminated in water vapor and carbon dioxide. Examples of incorporation are exchange with labile hydrogen in protein and labile oxygen in phosphate and carboxyl. Thus, the isotope dilution space of ^2^H is on average 4%, and the isotope dilution space of ^18^O is on average 1% larger than the total body water volume (Schoeller et al. 1980). Fractionation occurs, because heavy isotopes vapourize less readily and thus water vapour as lost through skin and breath is slightly less enriched for ^2^H and ^18^O than body water. In addition, carbon dioxide is about 4% more enriched for ^18^O than body water (Schoeller et al. 1986a). The generally adopted formula for the calculation of carbon dioxide production from apparent turnover rates of the hydrogen and oxygen of body water, with correction for incorporation and fractionation, is (Schoeller et al. 1986b):

$${\text{rCO}}_{2} ({\text{mol}}) = (N/ 2.0 7 8) \, ( 1.0 1K_{ 1 8} {-} 1.0 4 { }K_{ 2} ){-}0.0 2 4 6r_{\text{GF}} ,$$

where *r*_GF_ is the rate of water loss via fractionating gaseous routes and is estimated as 1.05 N (*K*_18_ − K_2_).

Validation studies were typically performed with simultaneously measurement of carbon dioxide production in a respiration chamber. Thus, the accuracy was shown to be within 2% with a coefficient of variation of 2–12% (Schoeller 1988; Speakman 1998). Validation studies included sedentary conditions as well as measurements at high-activity levels where subjects performed heavy bicycle ergometer work (Westerterp et al. 1988). The results demonstrated the utility of the doubly labelled water method for the determination of carbon dioxide production in the range of activity levels in daily life.

## Methodological aspects

Application of the doubly labelled method for the measurement of energy expenditure includes design of a protocol, preparation of the isotope dose, sample collection, sample analysis, and calculation of energy expenditure from the results obtained (Table 2). The most critical step is sample analysis, ideally performed in a dedicated laboratory with specific sample preparation systems and an isotope ratio mass spectrometer (Speakman 1997). One can set up a laboratory or send samples away to get them analyzed. There are commercial laboratories and some university laboratories performing analyses of external samples.

**Table 2:** Critical methodological aspects of the doubly labelled water method for the measurement of energy expenditure in humans

Observation interval	1 to 3 weeks
Isotope dose	>1.8 g water/kg body water with 10% ^18^O
	>0.12 g water/kg body water with 99% ^2^H
Sample collection	Background sample before dose
	Two independent samples directly after dose equilibration
	Two independent samples at the end of the observation interval
Sample analysis	Accuracy 0.5 ppm for both isotopes

With regard to the protocol, the optimal observation interval for the measurement of energy expenditure with doubly labelled water is one to three times the biological half-life of the isotopes (Lifson and McClintock 1966). The biological half-life of ^18^O ranges from 3 days in young children or extremely active adult subjects to about 10 days in very sedentary adult and old subjects (Westerterp 1999). In addition, measuring energy expenditure under daily living conditions with weekly cycles of physical activity routines, one often measures over multiples of a week. Thus, a typical observation interval is 1 week in children and endurance athletes, 2 weeks in adult subjects, and 3 weeks in the elderly.

With regard to the isotope dose, body water volume of the subject mainly determines the isotope dose. Larger subjects require more isotopes, as do men compared to similar mass women, because of their larger body water containing fat-free mass. The ultimate determinant of the dose is the final body water enrichment of the isotopes at the end of the observation interval, in combination with the variation in background enrichment and the analysis precision of body water samples. With the current sample analysis precision with isotope ratio mass spectrometry, subjects are dosed with at least 1.8 g water/kg body water of water with 10% ^18^O atoms and 0.12 g water/kg body water of water with 99% ^2^H atoms (International Atomic Energy Agency 2009). Thus, the initial background enrichment of body water for ^18^O of 2000 ppm is increased with 180 ppm and the initial background enrichment of body water for ^2^H of 150 ppm is increased with 120 ppm, or slightly higher values. The doubly labelled water method is considered safe for subjects of all ages, with no indications of health effects of the two isotopes at these concentrations (Leatherdale and Jones 1991).

With regard to sample collection, body water enrichment for ^18^O and ^2^H is measured in blood-, saliva-, or urine samples. Different body fluids are subject to different degrees of isotope fractionation and thus are not combined in the same protocol (Prentice 1990). Usually urine is chosen, where one should ensure that the urine has not been in the bladder for longer time, preventing accurate timing of the sample. A typical protocol starts with collection of a sample before isotope dose administration, to measure the background body water enrichment. The next sample is collected directly after dose administration and equilibration with the total body water. In healthy young adults, steady-state enrichment of the body water is reached around 2.5 h of ingestion of the isotope (Jankowski et al. 2004). Most studies allow a 4–6 h equilibration time. In larger subjects with a larger body water compartment, where equilibration takes longer, a protocol is adopted with overnight equilibration (Van Marken Lichtenbelt et al. 1994). In the simplest protocol, subsequent isotope elimination is determined by collecting one sample at the end of the observation interval, usually after 1–3 weeks, the two-point method.

Initially, it was preferred to calculate isotope elimination from serial samples, collected on a daily basis, the multipoint method (Cole and Coward 1992). Daily sampling improves the precision of the measurement because the results are calculated from more analyses, reducing the analytical variation by averaging (Schoeller et al. 1995). However, there might be substantial day-to-day differences in isotope elimination rate due to daily variation in energy expenditure. Nowadays, a protocol is indicated with at least two independent samples at the start and at the end of the observation interval. A protocol allowing for differences in equilibration time between subjects and for analytical variation is the ‘Maastricht protocol’ (Westerterp et al. 1995). The isotope dose is administered, after collecting a background sample, as a last consumption before the night. After overnight equilibration, two independent early morning samples are collected at the first day and with weekly intervals of the subsequent observation interval. Samples are preferably stored in glass vials with airtight caps to prevent isotope exchange through perfusion (Westerterp et al. 1995) and isotope fractionation through evaporation (Schoeller et al. 1986a).

**Fig. 2:**
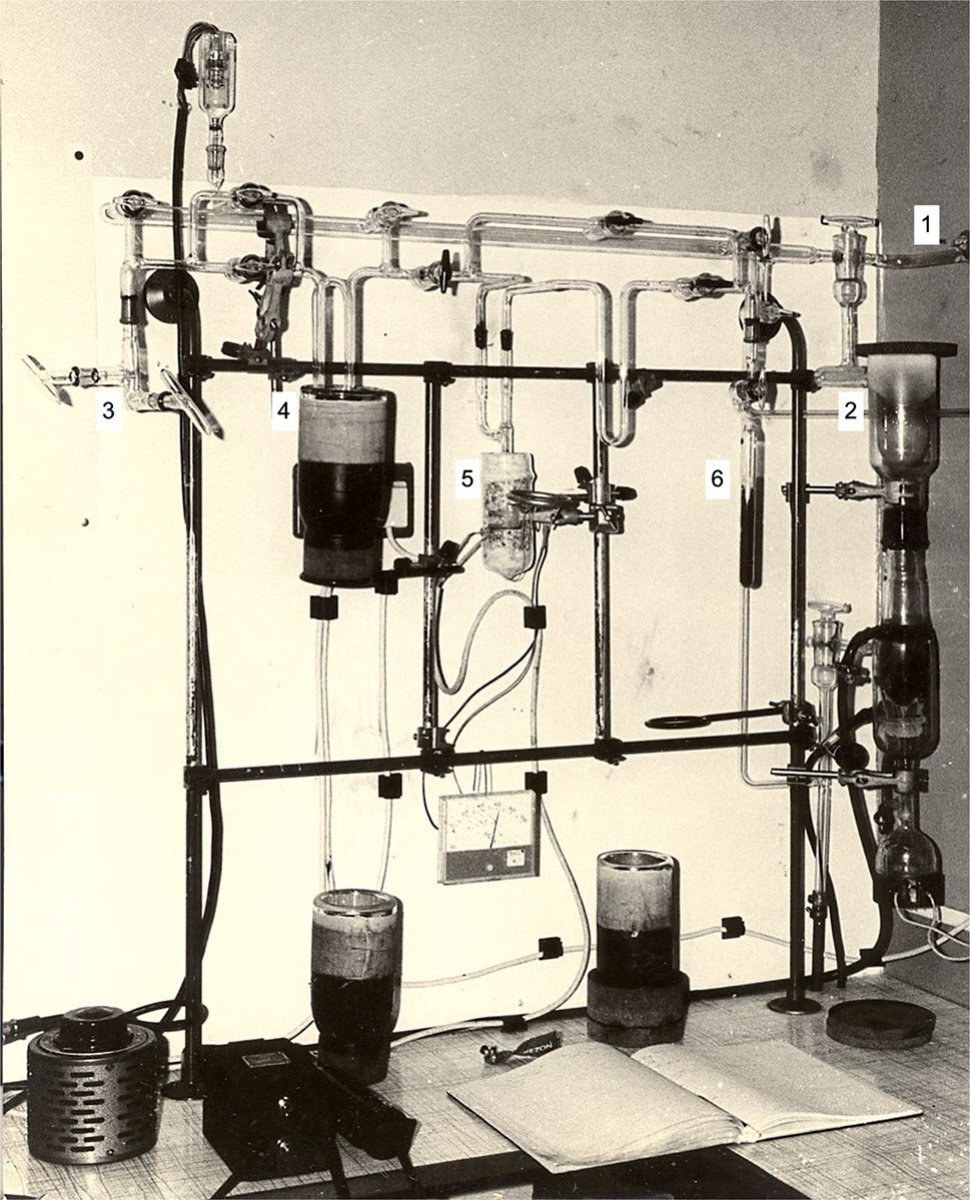
Preparation line for hydrogen gas samples at the Stable Isotope Geology Unit of the Scottish Universities Research and Reactor Centre in East Kilbride, Scotland 1977. The glass system is connected to a rotary pump (*1*) and a mercury diffusion pump (*2*) to create high vacuum. A blood-, saliva-, or urine sample in a sealed glass capillary is placed in a tube cracker (*3*), connected to the system and the system is pumped to vacuum. Then, the capillary is broken and water in the sample is frozen in a U-tube surrounded by liquid nitrogen in a dewar vessel (*4*). Subsequently, the frozen water is evaporated and passes a uranium furnace kept at 600 °C (*5*), where it is converted to hydrogen gas. The hydrogen gas is absorbed in a charcoal tube surrounded by liquid nitrogen in a dewar vessel (*6*). Finally, the charcoal tube with the sample is closed and transferred to an isotope ratio mass spectrometer for analysis

With regard to sample analysis, this is the most critical step in doubly labelled water assessment of energy expenditure. The isotopes are generally measured in the form of simple gases such a H_2_ and CO_2_. Water from blood-, saliva-, or urine samples is converted or equilibrated with H_2_ and with CO_2_, and the enrichment of the two gases is measured with isotope ratio mass spectrometry. As an example, Fig. 2 shows a preparation line for hydrogen, where water from blood-, saliva-, or urine samples is converted to hydrogen gas. Carbon dioxide for ^18^O analysis is typically prepared by equilibration. Water evaporated from a sample is frozen in an empty tube, an amount of carbon dioxide of known ^18^O is added, and equilibration takes place by leaving the sample water with the CO_2_ for several hours to a day at a fixed temperature between 25 and 40 °C. Since the start of the doubly labelled water method for the measurement of energy expenditure, sample preparation and analysis remains a dedicated and time-consuming procedure. In 1985, a rapid analytical technique for the determination of energy expenditure by the doubly labelled water method was described (Barrie and Coward 1985). An automated system was produced, for simultaneous analysis of ^2^H and ^18^O enrichment in body water samples, comprising two mass spectrometer analyzers (Aqua-Sira, VG-IsoGas, Middlewich, Cheshire, UK). The company sold at least seven systems, three to laboratories in the UK, and one in The Netherlands, France, Italy, and Australia, where only two laboratories got the system going and subsequently used it for some 15 years. The main bottleneck was a memory effect, because water introduced in the system is not completely removed when one sample is substituted for another. The memory results from adsorption of water on the walls of the system and in the uranium furnace. Nowadays, memory problems are still a bottleneck in sample analysis for doubly labelled water assessment of energy expenditure (Thorsen et al. 2011; Berman et al. 2012). Current methodology for sample analysis has an accuracy of at least 0.5 ppm for both isotopes (Wong and Clarke 2012, 2015), resulting in a coefficient of variation of the doubly labelled water method for the measurement of energy expenditure between 4 and 8% (Schoeller 1983).

With regard to calculation of energy expenditure, the doubly labelled water method measures carbon dioxide production, requiring an estimate of the energy equivalent of carbon dioxide for conversion to energy expenditure. The energy equivalent of carbon dioxide is a function of the substrate mixture being oxidized. It ranges from a minimum of 21.1 kJ/l for pure carbohydrate oxidation to 27.8 kJ/l for pure fat oxidation (Table 3). For a typical western diet, 55 energy% carbohydrate, 15 energy% protein, and 30 energy% fat, the energy equivalent of carbon dioxide is 23.5 kJ/l. Extreme values are 22.1 and 25.5 for a very low-fat diet, 75 energy% carbohydrate, 15 energy% protein, and 10 energy% fat, and a very high-fat diet, 25 energy% carbohydrate, 15 energy% protein, and 60 energy% fat, respectively. Measuring the respiratory quotient or predicting the respiratory quotient from the composition of the diet will result in negligible errors not exceeding ±2% (Black et al. 1986).

**Table 3:** Nutrients and resulting energy equivalents of oxygen and carbon dioxide when metabolized (Brouwer 1957)

Nutrient	Oxygen (kJ/l)	Carbon dioxide (kJ/l)
Carbohydrate	21.1	21.1
Protein	18.7	23.4
Fat	19.6	27.8

## Promise

The doubly labelled water method is the only method to measure energy expenditure in any environment, especially with regard to activity energy expenditure, where there is no interference with the behaviour of the subjects. Thus, the method is primarily applied for the measurement of the physical activity level of subjects. Additional applications include the assessment of energy requirement from total energy expenditure, validation of dietary assessment methods and validation of physical activity assessment methods with doubly labelled water measured energy expenditure as reference, and studies on body mass regulation with energy expenditure as a determinant of energy balance.

## Measuring physical activity level

**Fig. 3:**
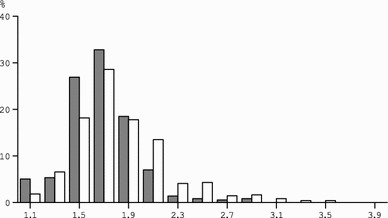
Frequency distribution of the value of the physical activity level, total energy expenditure as a multiple of resting energy expenditure, including all adult subjects with doubly labelled water-assessed energy expenditure in Maastricht until 2016: women (*N* = 358, *closed bars*) and men (*N* = 490, *open bars*)

Doubly labelled water assessment of energy expenditure is primarily applied for the measurement of the physical activity level of subjects. To compare the physical activity level within and between subjects, total energy expenditure is divided by resting energy expenditure, resulting in a figure without dimension: physical activity level = total energy expenditure/resting energy expenditure (FAO/WHO/UNU 2004). Dividing total energy expenditure by resting energy expenditure adjusts for differences in body size and composition. A larger subject has higher resting energy expenditure than a smaller subject. Total energy expenditure is higher as well, and divided by resting energy expenditure may result in a comparable physical activity level to a smaller subject. Data on doubly labelled water-assessed total energy expenditure show limits to physical activity level. In our site, data were compiled from studies performed since the start of the application in the 1980’s. The sample includes individuals aged 18 years or over, with a wide range for age, height, mass, and body mass index. Despite the wide variation in subject characteristics, there is a narrow range of physical activity level of the subjects (Fig. 3). The physical activity level for ‘sustained lifestyles’ ranges between 1.1–1.2 and 2.0–2.5 as suggested from earlier analyses (Black et al. 1996). Women and men show no difference in physical activity level. The minimum value of 1.1–1.2 is for a subject with no physical activity, total energy expenditure being the sum of basal metabolic rate, and diet-induced energy expenditure. The maximum value of 2.0–2.5 is determined by energy intake (Westerterp 1998). Higher values are difficult to maintain over a long period of time and generally result in mass loss, unless energy intake is supplemented. Physical activity level of a subject can be classified in three categories as defined by the last FAO/WHO/UNU expert consultation on human energy requirements (FAO/WHO/UNU 2004). The physical activity level value for sedentary and light activity lifestyles ranges between 1.40 and 1.69, for moderately active or active lifestyles between 1.70 and 1.99, and for vigorously active lifestyles between 2.00 and 2.40.

## Validation of dietary- and physical activity assessment methods

The doubly labelled water method has become the gold standard for the assessment of energy requirement (FAO/WHO/UNU 1985, 2004). Assessment of energy expenditure with doubly labelled water has demonstrated that self-report measures of food intake and physical activity are not accurate (Dhurandhar et al. 2015). There is not yet a method for the accurate determination of habitual dietary intake, and thus, energy requirement is derived from measured energy expenditure (Schoeller et al. 1990; Trabulsi and Schoeller 2001). Most studies show a lower value for reported energy intake compared with measured total energy expenditure. Underreporting of habitual intake can be explained by under-recording and under-eating (Goris et al. 2000; Goris and Westerterp 1999). In a clinical setting, the increased awareness of the nursing staff resulted in the opposite, overreporting of intake. Carefully collected energy intake data were inaccurate to such a degree as to be useless (Stallings et al. 1996). Reported intake remains not a suitable marker for energy requirement as evaluated with doubly labelled water (Lopes et al. 2016; Lins et al. 2016). Current development for improvement includes the use of micro-cameras to take pre- and post-meal photographs (Ptomey et al. 2015; Delisle Nyström et al. 2016; Pettitt et al. 2016). So far, doubly labelled water remains the reference for energy requirement, in healthy subjects as well as in patients (Prelack et al. 2017) and in athletes (Morehen et al. 2016).

Doubly labelled water measured energy expenditure not only is the current reference for energy requirement and improvement of dietary assessment methods but also for physical activity assessment methods.

New technology has resulted in a growing number of physical activity monitors to evaluate the activity level of an individual. The validity of activity monitors, as derived from studies using doubly labelled water-assessed activity energy expenditure as a reference, is not always sufficient. In a review on the performance of 11 different monitors, only three could explain more than 50% of the variation in activity-induced energy expenditure or physical activity level between subjects (Westerterp 2014). A more recent review confirmed that there is a large heterogeneity across studies in the performance of accelerometers to estimate activity-induced energy expenditure (Jeran et al. 2016). Most wearable devices do not yet provide a valid estimate of total energy expenditure (Murakami et al. 2016).

## Studies on body mass regulation

Doubly labelled water-assessed energy expenditure is a key parameter in studies on body mass regulation, where energy expenditure is a determinant of energy balance. The last decades, the prevalence of overweight and obesity has increased worldwide. Initially, it was suggested that modern inactive lifestyles were the predominant factor in the increasing prevalence of overweight and obesity (Prentice and Jebb 1995). The physical activity level and thus energy needs should have declined faster than energy intake as encouraged by the increasing availability of highly palatable foods. However, analysis of doubly labelled water-assessed physical activity level for trends over time showed that activity-induced energy expenditure has not declined over the same period that obesity rates have increased, and total energy expenditure of modern man is in line with energy expenditure in wild mammals (Westerterp and Speakman 2008). The relation between daily energy expenditure and body mass suggests that increase in energy intake has driven the increase in body mass (Swinburn et al. 2009). Physical activity level, as assessed with doubly labelled water, was shown to promote normal growth and accretion of fat-free mass (Butte et al. 2016). At adult age, an increase in physical activity level results only in modest mass loss or in some cases mass gain (Thomas et al. 2012).

Limitations of the doubly labelled water method for the measurement of energy expenditure are the length of the observation interval and the time-consuming sample analysis procedure. The observation interval ranges between 3 days in young children or extremely active adult subjects and 3 or 4 weeks in very sedentary and old subjects (Schoeller 1983; Westerterp 1999). Over shorter intervals, the change in isotope enrichment is too small to obtain a precise measure of the elimination rate. After longer intervals, the final enrichment cannot be measured precisely, getting too close to background values. Isotope analysis remains time-consuming, despite the development of on alternative for the traditional sample preparation and subsequent measurement of isotope enrichment with isotope ratio mass spectrometry. Immediate measurement of isotope enrichment in biological samples with laser absorption spectrometry can be performed in minutes (Thorsen et al. 2011; Berman et al. 2012). However, memory problems necessitate conditioning of the system by repeated sample injections, increasing the analysis time to 1 h for both isotopes. In addition, one cannot measure the initial samples with high enrichment and the final samples with low enrichment on the same day.

In conclusion, the doubly labelled water method for the measurement of total energy expenditure is a valuable addition to the spectrum of indirect calorimetry methods. It is the indicated method for the measurement of energy expenditure in unrestrained subjects in their normal surroundings. Then, the typical observation interval of one or more weeks can cover the regular cycle of activity routines or effects of exercise interventions on total energy expenditure.

## Abbreviations

FAO/WHO/UNU: Food and Agriculture Organization of the united nations/World Health Organization/United Nations University
*K*_2_: Elimination rate of heavy hydrogen
*K*_18_: Elimination rate of heavy oxygen
mol: Molecule
*N*: Number of body water molecules
ppm: Parts per million
^2^H: Heavy hydrogen
^18^O: Heavy oxygen
